# Association of Longer Leukocyte Telomere Length With Cardiac Size, Function, and Heart Failure

**DOI:** 10.1001/jamacardio.2023.2167

**Published:** 2023-07-26

**Authors:** Nay Aung, Qingning Wang, Stefan van Duijvenboden, Richard Burns, Svetlana Stoma, Zahra Raisi-Estabragh, Selda Ahmet, Elias Allara, Angela Wood, Emanuele Di Angelantonio, John Danesh, Patricia B. Munroe, Alistair Young, Nicholas C. Harvey, Veryan Codd, Christopher P. Nelson, Steffen E. Petersen, Nilesh J. Samani

**Affiliations:** 1 William Harvey Research Institute, Barts and The London School of Medicine and Dentistry, Queen Mary University of London, London, United Kingdom; 2 National Institute for Health and Care Research Barts Cardiovascular Biomedical Research Centre, Queen Mary University of London, London, United Kingdom; 3 Barts Heart Centre, St Bartholomew’s Hospital, Barts Health NHS Trust, West Smithfield, London, United Kingdom; 4 Department of Cardiovascular Sciences, University of Leicester and NIHR Leicester Biomedical Research Centre, Glenfield Hospital, Leicester, United Kingdom; 5 National Institute for Health and Care Research Leicester Biomedical Research Centre, Glenfield Hospital, Leicester, United Kingdom; 6 Nuffield Department of Population Health, University of Oxford, Oxford, United Kingdom; 7 School of Biomedical Engineering and Imaging Sciences, King’s College London, London, United Kingdom; 8 British Heart Foundation Cardiovascular Epidemiology Unit, Department of Public Health and Primary Care, University of Cambridge, Cambridge, United Kingdom; 9 Victor Phillip Dahdaleh Heart and Lung Research Institute, University of Cambridge, Cambridge, United Kingdom; 10 National Institute for Health and Care Research Blood and Transplant Research Unit in Donor Health and Genomics, University of Cambridge, Cambridge, United Kingdom; 11 British Heart Foundation Centre of Research Excellence, University of Cambridge, Cambridge, United Kingdom; 12 Health Data Research UK Cambridge, Wellcome Genome Campus and University of Cambridge, Cambridge, United Kingdom; 13 Cambridge Centre of Artificial Intelligence in Medicine, Cambridge, United Kingdom; 14 Health Data Science Centre, Human Technopole, Milan, Italy; 15 Department of Human Genetics, Wellcome Sanger Institute, Wellcome Genome Campus, Hinxton, United Kingdom; 16 MRC Lifecourse Epidemiology Centre, University of Southampton, Southampton, United Kingdom; 17 NIHR Southampton Biomedical Research Centre, University of Southampton and University Hospital Southampton NHS Foundation Trust, Southampton, United Kingdom

## Abstract

**Importance:**

Longer leukocyte telomere length (LTL) is associated with a lower risk of adverse cardiovascular outcomes. The extent to which variation in LTL is associated with intermediary cardiovascular phenotypes is unclear.

**Objective:**

To evaluate the associations between LTL and a diverse set of cardiovascular imaging phenotypes

**Design, Setting, and Participants:**

This is a population-based cross-sectional study of UK Biobank participants recruited from 2006 to 2010. LTL was measured using a quantitative polymerase chain reaction method. Cardiovascular measurements were derived from cardiovascular magnetic resonance using machine learning. The median (IQR) duration of follow-up was 12.0 (11.3-12.7) years. The associations of LTL with imaging measurements and incident heart failure (HF) were evaluated by multivariable regression models. Genetic associations between LTL and significantly associated traits were investigated by mendelian randomization. Data were analyzed from January to May 2023.

**Exposure:**

LTL.

**Main Outcomes and Measures:**

Cardiovascular imaging traits and HF.

**Results:**

Of 40 459 included participants, 19 529 (48.3%) were men, and the mean (SD) age was 55.1 (7.6) years. Longer LTL was independently associated with a pattern of positive cardiac remodeling (higher left ventricular mass, larger global ventricular size and volume, and higher ventricular and atrial stroke volumes) and a lower risk of incident HF (LTL fourth quartile vs first quartile: hazard ratio, 0.86; 95% CI, 0.81-0.91; *P* = 1.8 × 10^−6^). Mendelian randomization analysis suggested a potential causal association between LTL and left ventricular mass, global ventricular volume, and left ventricular stroke volume.

**Conclusions and Relevance:**

In this cross-sectional study, longer LTL was associated with a larger heart with better cardiac function in middle age, which could potentially explain the observed lower risk of incident HF.

## Key Points

**Question:**

Is leukocyte telomere length (LTL) associated with alterations in cardiovascular structure and function?

**Findings:**

In this cross-sectional study including 40 459 UK Biobank participants, longer LTL was associated with higher left ventricular mass, larger ventricular and atrial sizes, and higher stroke volumes. Mendelian randomization analysis demonstrated a potential causal genetic association between LTL and left ventricular mass, ventricular size, and left ventricular stroke volume, and longer LTL was associated with a lower risk of incident heart failure after accounting for potential confounders.

**Meaning:**

These findings highlight that modulation of LTL dynamics may have a role in improving cardiovascular structure and function, which could potentially explain the observed lower future risk of heart failure.

## Teaser

This cross-sectional study evaluates the associations between leukocyte telomere length and cardiac size, function, and heart failure.

## Introduction

Telomeres are protective caps at the end of chromosomes that progressively shorten with each cell division.^1,2,3^ When telomeres reach a critical length, cells enter senescence; hence, telomere length is a marker of cellular replicative capacity and history.^2^ At the population level, there is a considerable interindividual variation in mean telomere length, usually measured in leukocytes (leukocyte telomere length [LTL]) but also present in other tissues.^3^ In epidemiological studies, we and others have shown that shorter LTL is associated with risk of incident coronary artery disease (CAD) as well as heart failure (HF).^4,5,6^ Mendelian randomization (MR) analyses have strongly suggested that the association of shorter LTL with CAD is genetically causal, although evidence for an association with HF is less certain.^4^

Cardiac imaging measurements, such as left ventricular mass (LVM), are intermediary phenotypes whose variability has also been shown to influence adverse cardiovascular outcomes, including CAD and HF.^7^ Two previous studies have investigated the association of LTL with LVM.^8,9^ The first by Vasan et al^8^ investigated 850 Framingham Heart study participants, and the second by Kuznetsova and colleagues^9^ examined 334 volunteers from the Flemish Study on Environment, Genes and Health Outcomes. Both studies used the LVM estimated by measurements from M-mode echocardiography and reported a positive association between LTL and LVM. However, neither study examined whether the association was consistent with a potential causal effect.

UK Biobank (UKB) is a large population cohort established between 2006 and 2010 of participants aged 40 to 69 years at recruitment.^10^ Participants have been characterized in detail using questionnaires, physical measurements, urinary and plasma biomarker measurements, genomic assays, and longitudinal linkage with multiple health record systems.^11^ A subset of participants have also undergone cardiovascular magnetic resonance (CMR) scans. We have recently completed a large-scale measurement of LTL in UKB participants and identified a large number of genetic variants associated with LTL, which are useful for potential causal inference (MR analyses).^4,12^ We have also derived measurements of cardiac structure and function from the CMR scans using automated artificial intelligence–based protocols.^13,14^ Here, using these data sets, we have examined (1) observational associations between LTL and cardiac morphology, function, and geometry, including LVM, global ventricular volume and size, left ventricular stroke volume (LVSV), right ventricular stroke volume (RVSV), LVM to end-diastolic volume ratio (LVMVR), atrial maximum volume, and atrial emptying volume, (2) the genetic association between LTL and observationally associated CMR measurements using MR, and (3) the potential causal association between LTL and future development of HF.

## Methods

### Participants

**Figure 1.**
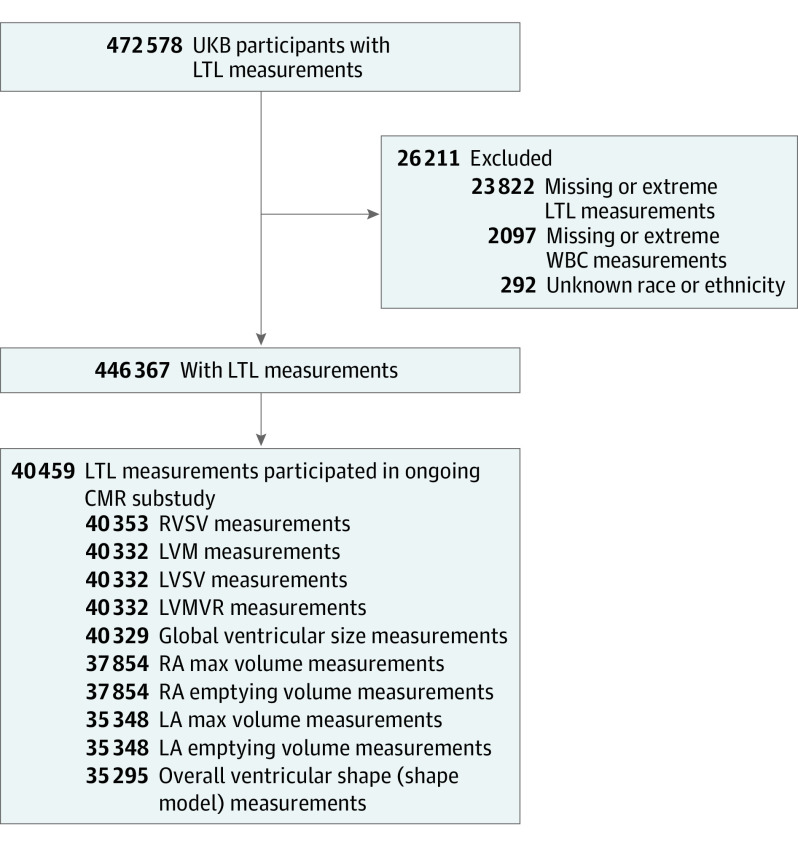
Sample Selection Flowchart CMR indicates cardiovascular magnetic resonance; LA, left atrial; LVM, left ventricular mass; LVMVR, left ventricle mass to end-diastolic volume ratio; LVSV, left ventricular stroke volume; LTL, leukocyte telomere length; RA, right atrial; RVSV, right ventricular stroke volume; UKB, UK Biobank; WBC, white blood cell count.

From participants with valid LTL measurements in UKB (n = 474 074),^12^ we excluded genetically related samples, randomly excluding 1 from each pair based on a kinship coefficient of K > 0.088 and samples with no genetic data or those that failed quality control. We also excluded participants who lacked information on race or ethnicity or white blood cell count, which are both associated with LTL^12^ and were used together with age and sex to adjust the trait associations. Race and ethnicity data were self-reported by the participants using a touchscreen questionnaire at the assessment center. Available options included Asian or Asian British, Black or Black British, Chinese, White, mixed race, other ethnic group, do not know, and prefer not to answer; those who selected do not know or prefer not to answer were excluded from the study sample. Among the remaining participants with LTL measurements (n = 446 367), 40 459 individuals participated in the ongoing CMR substudy (Figure 1). This study received the overall ethical approval for UKB studies from the NHS National Research Ethics Service on June 17, 2011, which was extended on June 18, 2021. All study participants provided written informed consent. This study followed the Strengthening the Reporting of Observational Studies in Epidemiology (STROBE) reporting guideline.

### Measurement of LTL

Details of LTL measurements, including extensive quality checks and the adjustment for technical factors in the UKB participants, have been described previously by our group.^12^ In brief, LTL was measured as the ratio of telomere repeat copy number (T) relative to that of a single copy gene (S) from the peripheral blood leukocyte DNA, extracted from blood collected at baseline, using a multiplex quantitative polymerase chain reaction method. LTL measurements (T/S ratios) were log_e_-transformed due to nonnormality (log_e_[LTL]) and *z* score standardized for all analyses (UKB field code: 22192).

### Derivation of CMR Parameters and Arterial Stiffness

Detailed CMR protocol and analysis methods have been described in prior publications.^13,14,15,16^ Of approximately 500 000 original UKB participants, those living within a reasonable traveling distance to 1 of the 4 imaging assessment centers were invited back for imaging enhancement substudy, with a target sample size of 100 000 individuals. The CMR scans available for the current study (n = 40 000) were obtained a mean (SD) 9.0 (1.7) years after the baseline visit. Segmentation of the left and right ventricular and atrial cavities and left ventricular myocardium were performed by automated machine learning algorithms, as detailed previously.^13^ Global ventricular volume was defined as the sum of the right and left ventricular end-diastolic volumes. LVSV, RVSV, and atrial stroke volumes were calculated by the difference between respective end-diastolic volume and end-systolic volume. LVMVR was derived by dividing LVM with left ventricular end-diastolic volume. End-diastolic biventricular shape models were obtained, as described previously.^17^ These models were compiled into a statistical shape atlas through principal component analysis, with principal components capturing the largest sources of variation in cardiac shape among the cohort. Through plotting these principal components, we can estimate biological features that they represent. The first principal component represents the overall size of the heart (higher scores having larger hearts), which was the greatest source of variation in heart shape among individuals (eFigure in Supplement 1).

### Statistical Analysis

The descriptive statistics are presented as means with SDs for continuous variables and counts with percentages for categorical variables. The trends across the LTL quartiles were examined by the Cuzick extension of the Wilcoxon rank sum test for continuous variables and the χ^2^ test for trend for ordinal variables. We removed the confounding influence of chronological age at baseline, white blood cell count, and self-reported race and ethnicity by taking the residuals of log_e_(LTL) regressed on these variables. Participants with missing data were excluded from the analysis. The associations between log_e_(LTL) residuals (independent variable) and CMR measures were evaluated in multivariable linear regression models adjusted for age at the imaging visit, sex, height, and weight. Significant associations were additionally adjusted for traditional cardiovascular risk factors (systolic blood pressure, diabetes, dyslipidemia, smoking status, and physical activity expressed in total metabolic equivalent of task minutes per week) to interrogate the potential confounding effects. Given the time lag between LTL sampling and CMR data acquisition, a sensitivity analysis investigating the interaction of the time lag between these 2 dates and LTL was conducted. We sought to identify the association between LTL and incident HF by performing survival analyses using Cox proportional hazards models adjusted for age, sex, body mass index, hypertension, hyperlipidemia, diabetes, and smoking status. We also explored the mediating effect of LTL and LVM on incident HF by introducing an interaction term. The effect sizes were represented by a 1-SD increase in log_e_(LTL) residuals. Significance was set at *P* < .05, and all *P* values were 2-tailed. All analyses were conducted in R version 4.0.2 (The R Foundation).

### MR Analysis

To investigate the potential causality and directionality of the associations of LTL with observationally associated imaging traits and with HF, we undertook an MR analysis, using large-scale genome-wide association study data sets.^4,14,18^ To assess whether the associations between LTL and imaging parameters and HF were consistent with a potential causal association, we used 130 conditionally independent, nonpleiotropic genetic variants that we have recently reported to be associated with LTL in UKB.^4^

For each analysis, we used the inverse variance–weighted MR method^19^ allowing for a random effect and also reported the *P* value for the intercept from MR Egger regression^20^ as a check for horizontal pleiotropy. As sensitivity analyses, we undertook MR analyses using the weighted median method,^21^ which is additionally robust in the presence of outliers, and the MR robust adjusted profile score method,^22^ which overcomes challenges related to measurement error, weak or invalid (due to pleiotropy) measurements, and selection bias (due to weak instrument). We also applied Steiger filtering implemented in the steiger_filtering() function in the R package TwoSampleMR to our genetic instruments, which removed variants that explain more variance in the outcome (ie, imaging measurements or HF) than the exposure (LTL) to minimize the risk of reverse causality. A combination of these approaches provides the best evidence for the presence of a genetic association consistent with a potential causal effect.

## Results

Of 40 459 included participants, 19 529 (48.3%) were men, and the mean (SD) age was 55.1 (7.6) years. The baseline characteristics of the study cohort stratified by LTL quartile are presented in Table 1, and characteristics stratified by sex are presented in eTables 1 and 2 in Supplement 1. Individuals in the higher LTL quartiles were more likely to be younger and female with a more favorable traditional cardiovascular risk profile. Most of the study cohort had CMR measurements within normal ranges^23^; the proportion of LVH was 2% (806 of 40 332). LVM was lower in higher LTL quartiles in the overall cohort, but when stratified by sex, LVM was higher in the higher LTL quartiles. Our study cohort of UKB participants who had CMR assessment were marginally younger, slightly more likely to be male and White, and had a lower prevalence of cardiometabolic risk factors than participants who did not receive CMR examination (eTable 3 in Supplement 1).

**Table 1.** Study Cohort Characteristics

Characteristic	Mean (SD)					*P* value for trend
	Full cohort	LTL quartile				
		1st	2nd	3rd	4th	
Total, No.	40 459	10 115	10 115	10 114	10 115	NA
Age at telomere visit, y	55.1 (7.6)	56.9 (7.4)	55.6 (7.4)	54.7 (7.5)	53.3 (7.5)	<.001
Age at imaging visit, y	64.2 (7.8)	65.8 (7.6)	64.6 (7.6)	63.8 (7.7)	62.4 (7.7)	<.001
Sex, No. (%)						
Female	20 930 (51.7)	4557 (45.1)	4983 (49.3)	5459 (54.0)	5931 (58.6)	<.001
Male	19 529 (48.3)	5558 (54.9)	5132 (50.7)	4655 (46.0)	4184 (41.4)	
Height, cm	169.2 (9.3)	169.6 (9.2)	169.3 (9.3)	169.1 (9.3)	168.7 (9.2)	<.001
Weight, kg	75.9 (15.0)	76.9 (15.0)	76.1 (15.1)	75.7 (15.1)	74.8 (15.0)	<.001
Race and ethnicity, No. (%)^a^						
Asian or Asian British	416 (1.0)	94 (0.9)	110 (1.1)	101 (1.0)	111 (1.1)	.35
Black or Black British	258 (0.6)	32 (0.3)	54 (0.5)	60 (0.6)	112 (1.1)	<.001
Chinese	115 (0.3)	25 (0.2)	16 (0.2)	25 (0.2)	49 (0.5)	<.001
White	39 277 (97.1)	9885 (97.7)	9852 (97.4)	9834 (97.2)	9706 (96.0)	<.001
Mixed race	189 (0.5)	40 (0.4)	40 (0.4)	46 (0.5)	63 (0.6)	.01
Other ethnic group	204 (0.5)	39 (0.4)	43 (0.4)	48 (0.5)	74 (0.7)	<.001
SBP, mm Hg	139.1 (18.7)	140.1 (18.7)	139.5 (18.5)	138.9 (18.6)	137.7 (18.8)	<.001
Diabetes, No. (%)	2359 (5.8)	731 (7.2)	624 (6.2)	523 (5.2)	481 (4.8)	<.001
Hyperlipidemia, No. (%)	14191 (35.1)	3920 (38.8)	3696 (36.5)	3447 (34.1)	3128 (30.9)	<.001
Active smoker, No. (%)	1323 (6.4)	328 (6.2)	345 (6.7)	345 (6.7)	305 (5.9)	.54
Physical activity (total MET min per wk)	2750.4 (2432.8)	2706.1 (2408.5)	2796.3 (2474.2)	2759.4 (2441.1)	2740.1 (2406.7)	.20
WBC, count/μL	6500 (1500)	6600 (1500)	6600 (1500)	6500 (1500)	6400 (1500)	<.001
LV mass, g	86.0 (22.4)	87.3 (22.3)	86.4 (22.5)	85.7 (22.4)	84.7 (22.3)	<.001
Indexed LV mass, g/m^2^	45.3 (8.6)	45.6 (8.7)	45.5 (8.7)	45.2 (8.5)	45.0 (8.6)	<.001
Global ventricular volume, mL	303.2 (68.6)	304.3 (68.0)	303.4 (68.7)	303.2 (68.9)	302.1 (68.8)	.001
Indexed global ventricular volume, mL/m^2^	160.6 (28.0)	159.8 (28.0)	160.5 (28.2)	160.8 (27.9)	161.3 (28.0)	<.001
LVMVR, g/mL	0.59 (0.09)	0.59 (0.09)	0.59 (0.09)	0.58 (0.09)	0.58 (0.09)	<.001
LVSV, mL	87.0 (19.2)	86.9 (19.0)	87.1 (19.3)	86.9 (19.3)	87.0 (19.0)	.43
Indexed LVSV, mL/m^2^	46.2 (8.5)	45.8 (8.4)	46.2 (8.6)	46.2 (8.5)	46.6 (8.4)	<.001
Overall ventricular size from shape model	0.01 (0.98)	0.05 (0.98)	0.03 (0.98)	0.01 (0.99)	−0.03 (0.98)	<.001
RVSV, mL	88.5 (20.2)	88.5 (20.2)	88.6 (20.1)	88.6 (20.4)	88.3 (20.0)	.17
Indexed RVSV, mL/m^2^	47.0 (8.9)	46.6 (9.0)	47.0 (8.9)	47.1 (9.0)	47.3 (8.8)	<.001
LA maximum volume, mL	44.3 (17.1)	44.5 (17.5)	44.2 (17.2)	44.4 (17.2)	44.2 (16.5)	.21
Indexed LA maximum volume, mL/m^2^	23.8 (9.1)	23.7 (9.2)	23.7 (9.2)	23.9 (9.2)	24.0 (8.9)	<.001
LA emptying volume, mL	28.0 (9.8)	27.8 (9.9)	27.9 (9.9)	28.1 (9.9)	28.1 (9.6)	<.001
Indexed LA emptying volume, mL/m^2^	15.1 (5.4)	14.8 (5.3)	15.0 (5.4)	15.2 (5.4)	15.3 (5.4)	<.001
RA maximum volume, mL	49.8 (20.6)	50.2 (21.4)	49.6 (20.0)	49.8 (20.8)	49.7 (20.2)	.68
Indexed RA maximum volume, mL/m^2^	26.9 (11.2)	26.8 (11.4)	26.7 (10.9)	26.9 (11.4)	27.0 (11.1)	.01
RA emptying volume, mL	24.4 (10.8)	24.3 (11.1)	24.3 (10.6)	24.4 (10.8)	24.5 (10.8)	.006
Indexed RA emptying volume, mL/m^2^	13.2 (6.0)	13.1 (6.1)	13.1 (5.9)	13.2 (6.1)	13.4 (6.1)	<.001

Abbreviations: LA, left atrium; LTL, leukocyte telomere length; LV, left ventricle; LVMVR, left ventricle mass to end-diastolic volume ratio; LVSV, left ventricle stroke volume; MET, metabolic equivalent of task; NA, not applicable; RA, right atrium; RVSV, right ventricular stroke volume; SBP, systolic blood pressure; WBC, white blood cell count.

SI conversion factor: To convert WBC to ×10^9^/L, multiply by 0.001.

^a^ Race and ethnicity data were self-reported by the participants using a touchscreen questionnaire at the assessment center. Available options included Asian or Asian British, Black or Black British, Chinese, White, mixed race, other ethnic group, do not know, and prefer not to answer; those who selected do not know or prefer not to answer were excluded from the study sample.

### Observational Associations Between LTL and Cardiovascular Measurements

After accounting for the differences in age, sex, height, and weight, a positive association was observed between LTL and LVM (β per 1-SD increment in log_e_[LTL] = 0.47; 95% CI, 0.34-0.60; *P* = 4.0 × 10^−12^) (Table 2). Similarly, longer LTL was associated with larger global ventricular volume (β = 1.33; 95% CI, 0.87-1.79; *P* = 1.8 × 10^−8^), larger overall ventricular size based on shape modeling (β = 0.01; 95% CI, 0.006-0.02; *P* = 1.2 × 10^−4^), higher LVSV (β = 0.35; 95% CI, 0.19-0.50; *P* = 8.7 × 10^−6^), higher RVSV (β = 0.34; 95% CI, 0.18-0.50; *P* = 3.2 × 10^−5^), larger left atrial maximal volume (β = 0.23; 95% CI, 0.05-0.41; *P* = .01), and higher left atrial emptying volume (β = 0.12; 95% CI, 0.02-0.23; *P* = .02). Additional adjustment with cardiovascular risk factors (systolic blood pressure, diabetes, dyslipidemia, smoking status, and physical activity level) slightly attenuated the effect sizes while retaining the statistical significance (Table 2). In contrast, there were no significant associations of LTL with LVMVR, an adverse remodeling phenotype, after adjusting for age, sex, height, and weight. A sensitivity analysis investigating the interaction between LTL and the time lag (between LTL sampling date and imaging visit date) did not find any significant results.

**Table 2.** Multivariable Regression Results for the Association Between Leukocyte Telomere Length and Cardiovascular Measurements

Measure	Model 1^a^		Model 2^b^	
	β (95% CI)	*P* value	β (95% CI)	*P* value
LV mass, g	0.47 (0.34 to 0.60)	3.97 × 10^−12^	0.37 (0.24 to 0.50)	2.31 × 10^−8^
Global ventricular volume, mL	1.33 (0.87 to 1.79)	1.84 × 10^−8^	1.17 (0.70 to 1.63)	9.37 × 10^−7^
Overall ventricular size from shape model	0.01 (0.006 to 0.02)	1.23 × 10^−4^	0.01 (0.004 to 0.02)	7.98 × 10^−4^
LVSV, mL	0.35 (0.19 to 0.50)	8.67 × 10^−6^	0.30 (0.15 to 0.46)	1.26 × 10^−4^
LVMVR, g/mL	6.90 × 10^−4^ (−1.05 × 10^−4^ to 1.49 × 10^−3^)	.09	NA	NA
RVSV, mL	0.34 (0.18 to 0.50)	3.15 × 10^−5^	0.27 (0.11 to 0.43)	8.76 × 10^−4^
LA maximum volume, mL	0.23 (0.05 to 0.41)	.01	0.19 (0.01 to 0.38)	.04
LA emptying volume, mL	0.12 (0.02 to 0.23)	.02	0.09 (−0.01 to 0.20)	.09
RA maximum volume, mL	0.15 (−0.06 to 0.35)	.16	NA	NA
RA emptying volume, mL	0.09 (−0.02 to 0.20)	.09	NA	NA

Abbreviations: LA, left atrium; LV, left ventricle; LVMVR, left ventricle mass to end-diastolic volume ratio; LVSV, left ventricle stroke volume; NA, not applicable; RA, right atrium; RVSV, right ventricular stroke volume.

^a^ Model 1 was adjusted for age, sex, height, and weight.

^b^ Model 2 was adjusted for age, sex, height, weight, systolic blood pressure, diabetes, hyperlipidemia, current smoking, and total metabolic equivalent of task minutes per week.

### Longitudinal Association Between LTL and Incident HF

**Figure 2.**
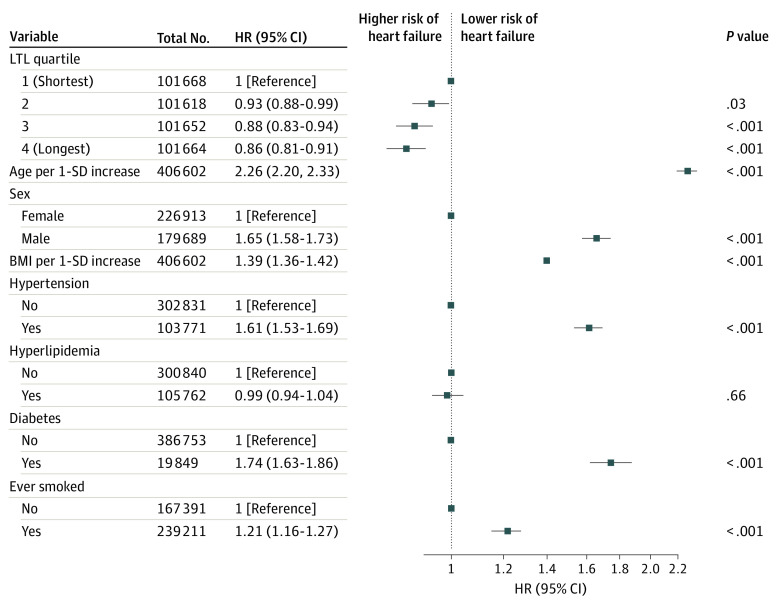
Longitudinal Association Between Leukocyte Telomere Length (LTL) and Incident Heart Failure BMI indicates body mass index; HR, hazard ratio.

Among 406 602 UKB participants with valid LTL measurements free from prevalent cardiovascular diseases, 7827 individuals had incident HF over a median (IQR) follow-up of 12.0 (11.3-12.7) years. In Cox proportional hazards analysis adjusted for age, sex, and other cardiovascular risk factors, longer LTL was associated with a lower future risk of HF (LTL fourth quartile vs first quartile: hazard ratio, 0.86; 95% CI, 0.81-0.91; *P* = 1.8 × 10^−6^) (Figure 2). Formal mediation analysis of LTL on the association between LVM (or other imaging traits) and HF was not feasible due to the low event rates in the CMR subcohort (approximately 100 events among approximately 40 000 participants) at this stage. Our exploratory interaction analysis of LTL and LVM on incident HF showed an association with lower risk (interaction HR, 0.87; 95% CI, 0.76-0.99; *P* = .04).

### MR Analyses

**Figure 3.**
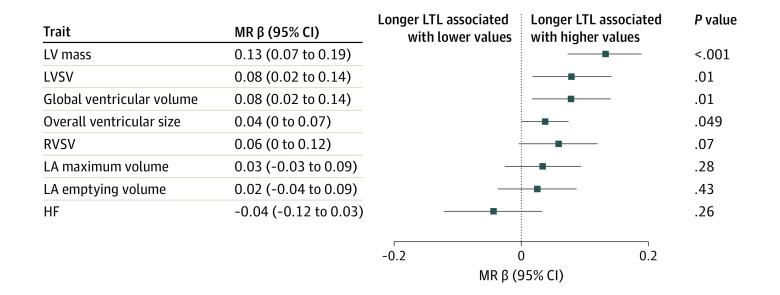
Mendelian Randomization Associations Between Leukocyte Telomere Length (LTL) and Cardiac Imaging Traits and Heart Failure (HF) LA, left atrial; LV indicates left ventricular; LVSV, left ventricular stroke volume; MR, mendelian randomization; RVSV, right ventricular stroke volume.

Using 130 genetic variants independently associated with LTL as instruments (eTable 4 in Supplement 1), we observed genetic associations of LTL with LVM (β = 0.13; 95% CI, 0.07-0.19; *P* = .0001), LVSV (β = 0.08; 95% CI, 0.02-0.14; *P* = .01), global ventricular volume (β = 0.08; 95% CI, 0.02-0.14; *P* = .01), and biventricular overall size (β = 0.04; 95% CI, 0.0002-0.07; *P* = .049) from shape model with inverse variance–weighted MR (Figure 3). Other imaging traits and HF did not achieve a statistically significant association with LTL, although the overall effect directions were concordant with observational results. There was no evidence of confounding by directional horizontal pleiotropy. Sensitivity analyses with the weighted median and MR robust adjusted profile score methods gave similar estimates as our primary inverse variance–weighted MR models. Furthermore, Steiger filtering, which removed genetic variants that explain more variance in the outcomes, did not materially alter our findings (eTable 5 in Supplement 1).

## Discussion

To our knowledge, this is the first and largest study to investigate the potential causal association between LTL and a comprehensive set of cardiac structure and function, robustly measured with CMR. Our principal findings are that in a middle-aged population (1) longer LTL was associated with higher LVM, larger global ventricular volume and overall size, and higher ventricular and atrial stroke volumes; (2) longer LTL was associated with a lower risk of incident HF even after accounting from traditional cardiovascular risk factors; and (3) the genetic associations between LTL and LVM, LVSV, and global ventricular volume are concordant with the observational results.

Our findings of an association of longer LTL with increased LVM are consistent with 2 previous reports^8,9^ that assessed LVM using echocardiography and build on these findings. We advanced this insight by highlighting that longer LTL was also associated with larger global ventricular volume and size and higher LVSV. Our finding of better left ventricular systolic function with longer LTL in a general population parallels the data from 2 small prior studies, which reported the associations between shorter LTL and reduced left ventricular ejection fraction in a hypertensive mouse model^24^ and in a human HF cohort.^25^ The overall pattern of cardiac morphofunctional differences observed with longer LTL (higher LVM, larger global ventricular volume, static LVMVR, larger atria, and higher ventricular and atrial stroke volumes) closely resembles beneficial balanced myocardial remodeling frequently seen with the physiological adaptation to exercise (eg, athlete heart).^26^ We also provide compelling genetic evidence, based on multiple MR approaches, that the associations of LTL with LVM, global ventricular volume, and LVSV are consistent with a potential causal association.

The impact of LTL on cardiac structure and function could have clinical relevance. We demonstrated in this work that longer LTL was associated with a reduced observed incidence of HF in UKB (hazard ratio, 0.86; 95% CI, 0.81-0.91; *P* = 1.8 × 10^−6^). The MR analysis was nonsignificant (odds ratio per 1-SD longer LTL, 0.96; 95% CI, 0.89-1.03), possibly related to low power. However, no firm conclusion can be drawn based on these data, and future studies using information from larger genome-wide association study are needed. Other studies have shown that LTL is shorter in patients with HF and is associated with poor prognosis.^6,27,28,29^ Experimental studies also directly support a role of telomere dynamics in cardiac structure and function. With aging, telomerase knockout mice hearts showed shortening of telomeres, attenuated proliferation and increased apoptosis of cardiomyocytes, and greater cardiac remodeling and left ventricular failure.^30,31^ On the other hand, enhanced expression of telomerase reverse transcriptase in rat cardiomyocytes preserved telomere length and induced cardiomyocyte proliferation, hypertrophy, and survival.^32^ While it is recognized that left ventricular hypertrophy and left ventricular dilatation in isolation are associated with adverse outcomes, through access to a more comprehensive set of imaging features, our study demonstrated a more global positive pattern of cardiac remodeling in association with longer LTL, which could explain the lower incidence of HF.

### Strengths and Limitations

Our study benefited from several important advantages, including (1) access to the largest sample size to date of LTL data with diverse and accurate cardiovascular imaging measurements using the reference-standard CMR and (2) application of MR for potential causal inference analysis using data from recent large genome-wide association studies. Nevertheless, several limitations need to be acknowledged. First, there is a healthy volunteer selection bias in the UKB, with the participants being older, more affluent, and having a healthier lifestyle with fewer comorbid conditions than the UK general population.^33^ The imaging substudy cohort is even slightly healthier than the overall UKB cohort. In line with this observation, most of our study cohort had imaging measurements within normal physiological ranges, and the applicability of our findings in disease states leading to left ventricular hypertrophy is uncertain. Second, most of our cohort (97%) is White, which may limit the generalizability of our findings in underrepresented races and ethnicities. Third, telomere length was quantified in blood leukocytes, which may not reflect cell-specific or tissue-specific telomere length. Fourth, the LTL and CMR measurements were obtained at different time points. The impact of this on the findings is uncertain but, if anything, is likely to have blunted the magnitude of the observed associations. Furthermore, our findings from MR, which circumvents the issues of confounding, measurement errors, and reverse causation in observational studies, provide concordant results for the key findings.

## Conclusions

In this study, longer LTL was associated with higher LVM, larger global ventricular size, and better cardiac function and a lower risk of incident HF. Further investigations into the prognostic relevance of LTL in adverse cardiac remodeling and the related mechanistic pathways could provide insights into the novel risk stratification approaches and therapeutic targets for HF.

## Notes

Supplement 1. **eFigure.** First Principal Component (PC1) Plot of Biventricular Diffeomorphic Subdivision Mesh Models Constructed at End-Diastole **eTable 1.** Study Cohort Characteristics for Men **eTable 2.** Study Cohort Characteristics for Women **eTable 3.** Baseline Characteristics of UK Biobank Participants With or Without CMR Assessment **eTable 4.** LTL Genetic Variants Used in Mendelian Randomisation **eTable 5.** Mendelian Randomisation Results for the Association Between LTL and Cardiovascular Measurements and Heart Failure

Supplement 2. Data Sharing Statement
