# Supplementary material for: Association of Longer Leukocyte Telomere Length With Cardiac Size, Function, and Heart Failure
Source: JAMA Cardiol. 2023 Jul 26;8(9):808–15. doi: 10.1001/jamacardio.2023.2167 (PMC10372756; doi:10.1001/jamacardio.2023.2167)
Supplement: Supplement 1. — eFigure. First Principal Component (PC1) Plot of Biventricular Diffeomorphic Subdivision Mesh Models Constructed at End-Diastole eTable 1. Study Cohort Characteristics for Men eTable 2. Study Cohort Characteristics for Women eTable 3. Baseline Characteristics of UK Biobank Participants With or Without CMR Assessment eTable 4. LTL Genetic Variants Used in Mendelian Randomisation eTable 5. Mendelian Randomisation Results for the Association Between LTL and Cardiovascular Measurements and Heart Failure [file jamacardiol-e232167-s001.pdf]

Supplemental Online Content

Aung N, Wang Q, van Duijvenboden S, et al. Association of longer leukocyte telomere length with cardiac size, function, and heart failure. *JAMA Cardiol*. Published online July 26, 2023. doi:10.1001/jamacardio.2023.2167

eFigure. First Principal Component (PC1) Plot of Biventricular Diffeomorphic Subdivision Mesh Models Constructed at End-Diastole
eTable 1. Study Cohort Characteristics for Men
eTable 2. Study Cohort Characteristics for Women
eTable 3. Baseline Characteristics of UK Biobank Participants With or Without CMR Assessment
eTable 4. LTL Genetic Variants Used in Mendelian Randomisation
eTable 5. Mendelian Randomisation Results for the Association Between LTL and Cardiovascular Measurements and Heart Failure

This supplementary material has been provided by the authors to give readers additional information about their work.

© 2023 Aung N et al. *JAMA Cardiology*.

eFigure. First Principal Component (PC1) Plot of Biventricular Diffeomorphic Subdivision Mesh Models Constructed at End-Diastole

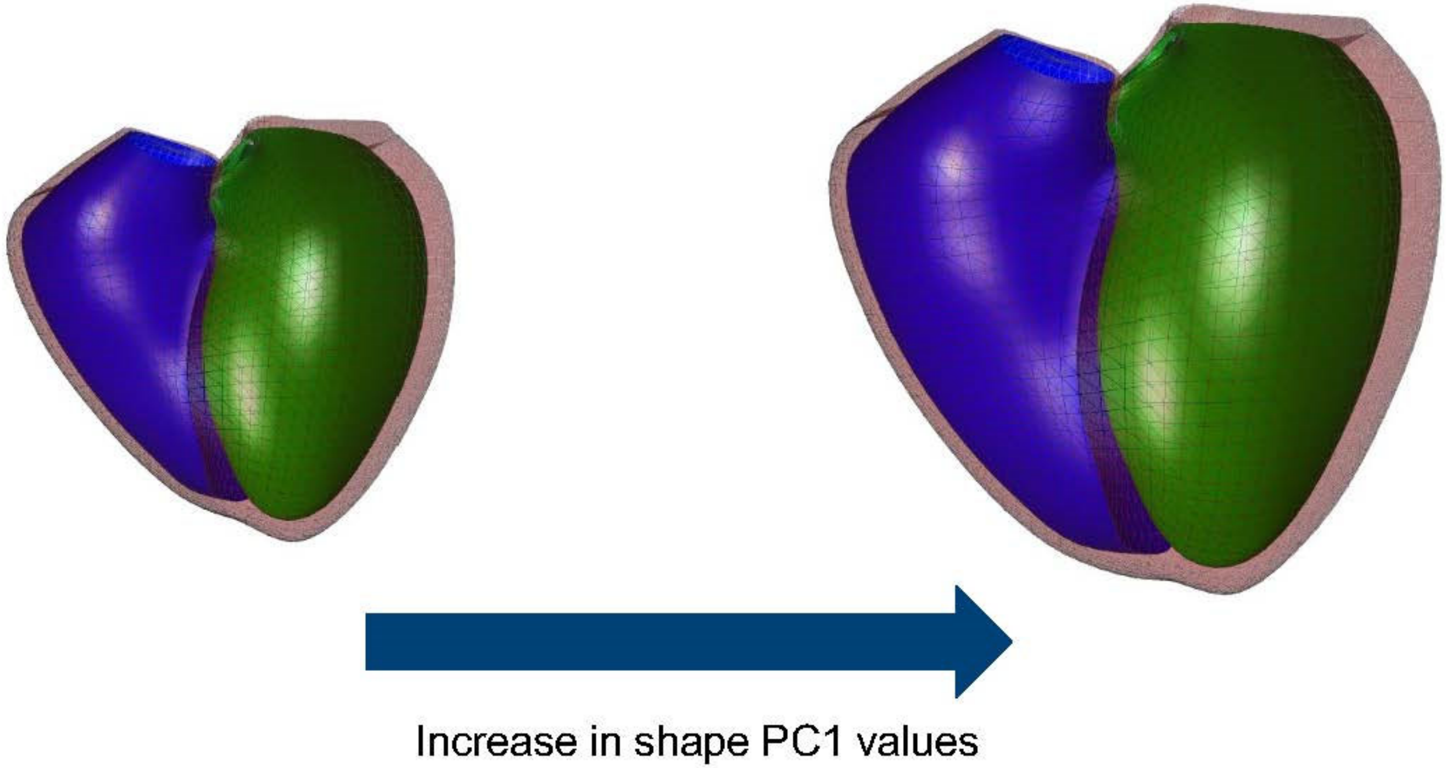

The figures were plotted at -2 and +2 standard deviations from the mean shape representing overall heart shape (left ventricle: green, right ventricle: blue, myocardium: pink).

© 2023 Aung N et al. *JAMA Cardiology*.

eTable 1. Study Cohort Characteristics for Men

		LTL quartiles				
	**Full cohort**	**1st**	**2nd**	**3rd**	**4th**	**P trend**
N	19529	5558	5132	4655	4184	
Age telomere visit (year)	55.81 (7.66)	57.76 (7.25)	56.13 (7.50)	55.16 (7.63)	53.57 (7.71)	<0.0001
Age imaging visit (year)	64.83 (7.84)	66.71 (7.46)	65.13 (7.71)	64.23 (7.82)	62.62 (7.89)	<0.0001
Height (cm)	176.06 (6.63)	175.55 (6.59)	175.92 (6.59)	176.36 (6.62)	176.60 (6.68)	<0.0001
Weight (kg)	83.50 (13.30)	83.31 (13.28)	83.30 (13.42)	83.87 (13.47)	83.57 (13.00)	0.0324
Ethnicity						
Asian	271 (1.4)	65 (1.2)	74 (1.4)	65 (1.4)	67 (1.6)	0.094
Black	128 (0.7)	21 (0.4)	29 (0.6)	34 (0.7)	44 (1.1)	<0.0001
Chinese	44 (0.2)	13 (0.2)	7 (0.1)	9 (0.2)	15 (0.4)	0.2122
Mixed	63 (0.3)	16 (0.3)	11 (0.2)	17 (0.4)	19 (0.5)	0.0901
Other	82 (0.4)	17 (0.3)	15 (0.3)	27 (0.6)	23 (0.5)	0.014
White	18941 (97.0)	5426 (97.6)	4996 (97.3)	4503 (96.7)	4016 (96.0)	<0.0001
SBP (mmHg)	141.76 (17.33)	142.39 (17.66)	142.12 (17.32)	141.61 (17.02)	140.66 (17.18)	<0.0001
Diabetes mellitus	1511 (7.7)	506 (9.1)	416 (8.1)	325 (7.0)	264 (6.3)	<0.0001
Hyperlipidaemia	8116 (41.6)	2508 (45.1)	2178 (42.4)	1872 (40.2)	1558 (37.2)	<0.0001
Smoking status	763 (7.6)	205 (7.1)	204 (7.8)	192 (8.0)	162 (7.5)	0.4641
Physical activity (Total MET minutes per week)	2778.28 (2475.19)	2753.27 (2475.08)	2805.55 (2497.08)	2767.30 (2466.19)	2790.33 (2458.98)	0.2443
WBC ($\times 10^9$/L)	6.50 (1.48)	6.60 (1.48)	6.56 (1.45)	6.43 (1.50)	6.37 (1.48)	<0.0001
LV mass (g)	102.38 (18.79)	101.17 (18.50)	101.86 (18.81)	103.06 (18.84)	103.87 (18.97)	<0.0001
Indexed LV mass (g/m^2)	50.75 (7.82)	50.28 (7.74)	50.57 (7.88)	50.93 (7.73)	51.37 (7.88)	<0.0001
Global ventricular volume (ml)	347.25 (62.33)	341.47 (61.27)	345.05 (62.28)	350.33 (62.54)	354.18 (62.71)	<0.0001
Indexed global ventricular volume (ml)	172.59 (28.85)	170.16 (28.46)	171.83 (29.10)	173.61 (28.67)	175.63 (28.91)	<0.0001
Overall ventricular size from shape model	0.75 (0.74)	0.69 (0.73)	0.73 (0.74)	0.79 (0.75)	0.82 (0.73)	<0.0001

© 2023 Aung N et al. *JAMA Cardiology*.

LVMVR (g/ml)	0.62 (0.09)	0.62 (0.09)	0.62 (0.09)	0.62 (0.09)	0.62 (0.09)	0.0077
LVSV (ml)	96.23 (19.27)	94.66 (18.70)	95.89 (19.41)	96.77 (19.66)	98.11 (19.23)	<0.0001
Indexed LVSV (ml/m^2)	47.85 (9.16)	47.20 (8.96)	47.78 (9.33)	47.96 (9.22)	48.66 (9.07)	<0.0001
RVSV (ml)	98.80 (19.96)	97.27 (19.82)	98.24 (19.82)	99.78 (20.21)	100.46 (19.84)	<0.0001
Indexed RVSV (ml/m^2)	49.14 (9.54)	48.51 (9.56)	48.95 (9.52)	49.47 (9.54)	49.85 (9.47)	<0.0001
LA maximum volume (ml)	45.63 (19.39)	45.88 (19.67)	45.56 (19.46)	45.41 (19.33)	45.63 (19.01)	0.96
Indexed LA maximum volume (ml/m^2)	22.77 (9.63)	22.93 (9.74)	22.79 (9.71)	22.58 (9.55)	22.75 (9.48)	0.3736
LA emptying volume (ml)	28.24 (11.07)	28.14 (11.05)	28.28 (11.27)	28.17 (11.04)	28.38 (10.88)	0.1561
Indexed LA emptying volume (ml/m^2)	14.12 (5.57)	14.09 (5.56)	14.17 (5.70)	14.04 (5.54)	14.17 (5.48)	0.6072
RA maximum volume (ml)	53.38 (23.72)	53.58 (24.37)	53.00 (22.81)	53.31 (23.95)	53.63 (23.71)	0.4175
Indexed RA maximum volume (ml/m^2)	26.81 (12.24)	26.96 (12.52)	26.68 (11.86)	26.73 (12.39)	26.87 (12.17)	0.9462
RA emptying volume (ml)	24.75 (12.02)	24.79 (12.25)	24.74 (11.69)	24.62 (11.88)	24.84 (12.29)	0.6285
Indexed RA emptying volume (ml/m^2)	12.43 (6.18)	12.48 (6.28)	12.46 (6.04)	12.35 (6.12)	12.45 (6.28)	0.804

SBP, systolic blood pressure; MET, metabolic equivalent of task; WBC, white blood cell count; LV, left ventricle; LVMVR, LV mass to end-diastolic volume ratio; LVSV, LV stroke volume; RVSV, right ventricular stroke volume; LA, left atrium; RA, right atrium; Other ethnicity category refers participants who selected "Other ethnic group" in the self-reported questionnaire.

© 2023 Aung N et al. *JAMA Cardiology*.

eTable 2. Study Cohort Characteristics for Women

	**Full cohort**	**1st**	**2nd**	**3rd**	**4th**	**P trend**
N	20930	4557	4983	5459	5931	
Age telomere visit (year)	54.49 (7.42)	55.85 (7.37)	55.07 (7.35)	54.30 (7.35)	53.15 (7.34)	<0.0001
Age imaging visit (year)	63.50 (7.60)	64.75 (7.58)	64.05 (7.54)	63.38 (7.56)	62.18 (7.50)	<0.0001
Height (cm)	162.76 (6.27)	162.43 (6.24)	162.49 (6.28)	162.94 (6.36)	163.08 (6.18)	<0.0001
Weight (kg)	68.78 (12.96)	69.16 (13.14)	68.58 (12.85)	68.77 (12.79)	68.67 (13.06)	0.14
Ethnicity						
Asian	145 (0.7)	29 (0.6)	36 (0.7)	36 (0.7)	44 (0.7)	0.6228
Black	130 (0.6)	11 (0.2)	25 (0.5)	26 (0.5)	68 (1.1)	<0.0001
Chinese	71 (0.3)	12 (0.3)	9 (0.2)	16 (0.3)	34 (0.6)	0.0022
Mixed	126 (0.6)	24 (0.5)	29 (0.6)	29 (0.5)	44 (0.7)	0.1947
Other	122 (0.6)	22 (0.5)	28 (0.6)	21 (0.4)	51 (0.9)	0.0301
White	20336 (97.2)	4459 (97.8)	4856 (97.5)	5331 (97.7)	5690 (95.9)	<0.0001
SBP (mmHg)	136.52 (19.48)	137.28 (19.44)	136.79 (19.24)	136.58 (19.56)	135.68 (19.60)	<0.0001
Diabetes mellitus	848 (4.1)	225 (4.9)	208 (4.2)	198 (3.6)	217 (3.7)	5e-04
Hyperlipidaemia	6075 (29.0)	1412 (31.0)	1518 (30.5)	1575 (28.9)	1570 (26.5)	<0.0001
Smoking status	560 (5.2)	123 (5.2)	141 (5.5)	153 (5.5)	143 (4.8)	0.4528
Physical activity (Total MET minutes per week)	2723.85 (2391.54)	2647.55 (2322.03)	2786.54 (2450.14)	2752.42 (2419.16)	2703.91 (2367.83)	0.3581
WBC ($\times 10^9$/L)	6.52 (1.50)	6.59 (1.51)	6.53 (1.48)	6.51 (1.49)	6.45 (1.52)	<0.0001
LV mass (g)	70.77 (12.57)	70.27 (12.66)	70.53 (12.82)	70.98 (12.38)	71.17 (12.44)	<0.0001
Indexed LV mass (g/m^2)	40.27 (5.79)	39.91 (5.87)	40.21 (5.85)	40.37 (5.73)	40.50 (5.70)	<0.0001
Global ventricular volume (ml)	262.20 (44.51)	258.80 (43.95)	260.67 (44.36)	263.05 (44.32)	265.32 (45.01)	<0.0001
Indexed global ventricular volume (ml)	149.46 (22.00)	147.25 (21.51)	148.90 (21.75)	149.86 (22.01)	151.27 (22.42)	<0.0001
Overall ventricular size from shape model	-0.66 (0.63)	-0.71 (0.63)	-0.68 (0.63)	-0.65 (0.62)	-0.62 (0.63)	<0.0001
LVMVR (g/ml)	0.56 (0.08)	0.56 (0.08)	0.56 (0.08)	0.56 (0.08)	0.55 (0.07)	<0.0001

© 2023 Aung N et al. *JAMA Cardiology*.

LVSV (ml)	78.36 (14.44)	77.48 (14.42)	78.11 (14.40)	78.41 (14.43)	79.19 (14.46)	<0.0001
Indexed LVSV (ml/m^2)	44.69 (7.46)	44.10 (7.34)	44.63 (7.36)	44.69 (7.48)	45.19 (7.57)	<0.0001
RVSV (ml)	78.85 (14.92)	77.83 (14.80)	78.64 (14.78)	78.99 (14.99)	79.70 (15.02)	<0.0001
Indexed RVSV (ml/m^2)	44.98 (7.75)	44.30 (7.60)	44.95 (7.67)	45.03 (7.85)	45.46 (7.79)	<0.0001
LA maximum volume (ml)	43.16 (14.62)	42.77 (14.44)	42.90 (14.48)	43.60 (15.13)	43.27 (14.40)	0.0064
Indexed LA maximum volume (ml/m^2)	24.76 (8.53)	24.50 (8.39)	24.65 (8.48)	24.99 (8.79)	24.82 (8.45)	0.0141
LA emptying volume (ml)	27.76 (8.55)	27.36 (8.27)	27.58 (8.40)	28.02 (8.83)	27.96 (8.62)	<0.0001
Indexed LA emptying volume (ml/m^2)	15.94 (5.06)	15.69 (4.87)	15.87 (5.01)	16.08 (5.16)	16.06 (5.12)	3e-04
RA maximum volume (ml)	46.60 (16.62)	46.16 (16.32)	46.23 (15.97)	46.91 (17.30)	46.93 (16.72)	0.0169
Indexed RA maximum volume (ml/m^2)	26.89 (10.14)	26.61 (9.98)	26.72 (9.77)	27.06 (10.51)	27.09 (10.21)	0.0218
RA emptying volume (ml)	24.07 (9.59)	23.79 (9.44)	23.85 (9.35)	24.22 (9.85)	24.32 (9.65)	0.0019
Indexed RA emptying volume (ml/m^2)	13.90 (5.81)	13.72 (5.72)	13.79 (5.67)	13.98 (5.94)	14.05 (5.85)	0.0019

SBP, systolic blood pressure; MET, metabolic equivalent of task; WBC, white blood cell count; LV, left ventricle; LVMVR, LV mass to end-diastolic volume ratio; LVSV, LV stroke volume; RVSV, right ventricular stroke volume; LA, left atrium; RA, right atrium; Other ethnicity category refers participants who selected "Other ethnic group" in the self-reported questionnaire.

© 2023 Aung N et al. *JAMA Cardiology*.

eTable 3. Baseline Characteristics of UK Biobank Participants With or Without CMR Assessment

Variable	CMR-	CMR+	P value
N	406,097	40,459	<0.001
Age at baseline visit (year)	56.69 (8.12)	55.13 (7.56)	<0.001
Male sex	184955 (45.5)	19528 (48.3)	<0.001
Height (cm)	168.41 (9.28)	169.73 (9.17)	<0.001
Weight (kg)	78.13 (15.93)	76.69 (14.77)	<0.001
Ethnicity			<0.001
Asian	8144 (2.0)	416 (1.0)	
Black	6555 (1.6)	258 (0.6)	
Chinese	1278 (0.3)	115 (0.3)	
Mixed	2411 (0.6)	188 (0.5)	
Other	3760 (0.9)	204 (0.5)	
White	383890 (94.5)	39274 (97.1)	
SBP (mmHg)	138.12 (18.68)	135.26 (17.75)	<0.001
Diabetes mellitus	24568 (6.1)	1276 (3.2)	<0.001
Hyperlipidaemia	123206 (30.3)	9798 (24.2)	<0.001
Smoking status	241659 (59.7)	23153 (57.3)	<0.001
Physical activity (Total MET minutes per week)	2540.63 (2587.39)	2378.26 (2328.82)	<0.001
LTL (Adjusted T/S ratio)	0.83 (0.13)	0.84 (0.13)	<0.001

CMR, cardiovascular magnetic resonance; SBP, systolic blood pressure; LTL, leukocyte telomere length; Other ethnicity category refers participants who selected “Other ethnic group” in the self-reported questionnaire.

© 2023 Aung N et al. *JAMA Cardiology*.

eTable 4. LTL Genetic Variants Used in Mendelian Randomisation

Variant_rsID	Chromosome	Hg37_position	EA	NEA	Beta	SE	P
rs187540244	1	11224327	G	A	0.090569	0.01491	1.20E-09
rs66731853	1	20916238	G	A	0.0177791	0.00215421	1.50E-16
rs17185038	1	28219658	C	G	0.0257788	0.00405796	2.10E-10
rs6669563	1	32279629	G	A	-0.0182358	0.00202476	2.10E-19
1:41236837_CT_C	1	41236837	CT	C	-0.0145193	0.0024091	1.70E-09
rs41269079	1	45252015	T	A	-0.0153617	0.0025499	1.70E-09
rs139795227	1	92842367	A	C	-0.0599379	0.00873247	6.70E-12
rs4498805	1	110910397	G	T	-0.0150601	0.00200376	5.70E-14
rs3838300	1	114442355	C	CA	0.0325605	0.00260147	6.10E-36
rs11579626	1	146741960	A	C	-0.0265113	0.00357752	1.30E-13
rs61818036	1	151364199	G	A	0.0189441	0.00269589	2.10E-12
rs932002	1	226577306	C	T	0.0402052	0.00279667	7.30E-47
rs9752694	2	17874177	C	G	0.0142753	0.00204136	2.70E-12
rs56178008	2	29098543	T	A	-0.0143739	0.00201464	9.70E-13
rs202034370	2	54488018	T	TA	0.102784	0.00650108	2.60E-56
rs12613375	2	58984109	C	T	-0.017801	0.00290685	9.10E-10
rs775145631	2	210667432	TC	T	0.0274974	0.00203966	2.00E-41
rs35671754	2	216220870	G	T	-0.0124744	0.00219884	1.40E-08
rs869785	3	24347800	T	C	0.0147303	0.00212801	4.40E-12
rs575032615	3	47638657	A	G	-0.0614625	0.00862809	1.10E-12
rs78491606	3	72891547	A	C	0.0756311	0.00741168	1.90E-24
rs6776756	3	128215821	G	A	0.0174439	0.00203747	1.10E-17
3:138398778_TA_T	3	138398778	TA	T	-0.0145467	0.00203645	9.10E-13
rs41272947	3	160119525	G	A	0.0173328	0.00200757	5.90E-18
rs2293607	3	169482335	T	C	0.0944803	0.00233425	0.00E+00
rs753936006	4	2191750	CAAAAA	C	0.0250923	0.00324669	1.10E-14

© 2023 Aung N et al. *JAMA Cardiology*.

rs871134	4	7044380	C	T	0.0182986	0.0020263	1.70E-19
rs13129697	4	9926967	T	G	-0.0172326	0.00224134	1.50E-14
rs35500378	4	122729413	CACTT	C	0.014467	0.00205607	2.00E-12
rs4435700	4	164020174	C	A	-0.0535453	0.00237679	2.20E-112
rs112951499	5	50697	A	AAACCCT	-0.0338623	0.0043605	8.10E-15
rs7705526	5	1285974	C	A	-0.0776022	0.00216124	2.40E-282
rs61748181	5	1294166	C	T	0.059181	0.00595394	2.80E-23
5:78951569_GT_G	5	78951569	GT	G	0.0246769	0.00335566	1.90E-13
rs34255404	5	138935580	G	A	-0.0372615	0.00432318	6.80E-18
rs80324517	6	204031	G	A	-0.0396515	0.00466286	1.80E-17
rs117247304	7	69010	G	A	0.0636727	0.00734043	4.20E-18
rs13230646	7	23930316	T	C	0.0173277	0.00232377	8.90E-14
rs11769630	7	50257703	T	A	0.0256807	0.00389475	4.30E-11
rs2538745	7	76310784	T	C	0.012942	0.002056	3.10E-10
rs2056726	7	99780283	G	A	0.0228078	0.00243638	7.90E-21
rs609953	7	123422444	T	A	-0.0134807	0.00206753	7.00E-11
rs7790856	7	124459852	C	T	0.0437199	0.00220526	1.80E-87
rs4731541	7	128678236	C	G	0.0206119	0.00205962	1.40E-23
rs1985369	7	159119220	A	G	0.0311893	0.00300952	3.60E-25
rs2306646	8	21846586	G	C	0.0209417	0.00201898	3.30E-25
rs762679	8	48885436	T	A	-0.0310104	0.00285024	1.40E-27
rs564224004	8	56667353	C	CATATATTAT	0.0307332	0.0031563	2.10E-22
rs7012816	8	70964743	G	A	-0.0175855	0.00296549	3.00E-09
rs10112752	8	73958718	G	A	0.0287522	0.00202518	9.50E-46
rs540491189	8	74150379	G	T	-0.191486	0.0235116	3.80E-16
rs1023767	8	95530969	G	A	0.0183732	0.00234772	5.00E-15
rs4742448	9	826585	C	G	-0.0154567	0.00206109	6.40E-14
rs4743037	9	109639970	C	T	-0.0147971	0.00238094	5.10E-10
rs762222726	10	5816070	CAAACAT	C	0.0184706	0.00204789	1.90E-19

© 2023 Aung N et al. *JAMA Cardiology*.

rs12572897	10	96114835	G	A	0.0321488	0.0029776	3.60E-27
10:101274251_CT_C	10	101274251	CT	C	0.0223224	0.00205641	1.90E-27
rs4919611	10	103894939	C	A	0.0255935	0.00315632	5.10E-16
rs9419958	10	105675946	T	C	0.0810098	0.00293847	2.60E-167
rs939916	11	202253	G	A	-0.0241795	0.00216724	6.60E-29
rs10840270	11	9629553	C	G	-0.014383	0.00212494	1.30E-11
rs611646	11	108177097	T	A	0.0368309	0.00203547	3.50E-73
rs6590343	11	128500215	A	G	-0.0121739	0.00201444	1.50E-09
rs10845387	12	11757743	G	A	0.0141214	0.00209396	1.50E-11
rs12369950	12	24762109	T	C	0.0178308	0.00290205	8.00E-10
rs79977579	12	54694560	C	A	-0.0281517	0.00343182	2.30E-16
rs1907702	12	88955469	G	A	-0.0150247	0.00242651	5.90E-10
rs76666449	12	120904895	T	C	-0.0295125	0.00333186	8.20E-19
rs4758644	12	122943915	A	C	0.016736	0.00229018	2.70E-13
rs79228077	12	133046343	G	T	0.0150243	0.00224653	2.30E-11
rs1332941	13	41695100	A	G	-0.0256552	0.00273159	5.90E-21
rs35017269	13	73340177	G	A	-0.0673908	0.00816803	1.60E-16
rs3093888	14	20812951	G	A	0.028973	0.00452459	1.50E-10
rs73581419	14	21941148	C	T	-0.0229838	0.00324156	1.30E-12
rs12884911	14	65027871	C	T	0.0132878	0.00199829	2.90E-11
rs762810	14	65544367	C	A	0.0202965	0.00210014	4.30E-22
rs137901416	14	73418095	G	A	-0.04572	0.00332355	4.70E-43
14:91970514_GA_G	14	91970514	GA	G	-0.0192864	0.00200386	6.30E-22
rs1957937	14	96181360	A	T	-0.0209365	0.00273361	1.90E-14
rs17677991	15	42032383	C	G	-0.0222664	0.00210806	4.40E-26
rs181647350	15	50379219	T	C	0.0336811	0.00236985	7.70E-46
rs1980240	15	56774018	A	C	-0.0129625	0.0020416	2.20E-10
rs80116508	16	3650970	G	A	0.0352672	0.00415151	2.00E-17
rs11646283	16	9073060	T	C	-0.0153447	0.00203937	5.30E-14

© 2023 Aung N et al. *JAMA Cardiology*.

rs182059586	16	14652220	T	C	0.0571159	0.00680853	4.90E-17
rs450962	16	28413517	A	G	-0.0142833	0.00245428	5.90E-09
rs8053839	16	48390512	G	T	0.0139207	0.00204649	1.00E-11
rs12447324	16	50089038	C	A	-0.0168979	0.00256429	4.40E-11
rs76219171	16	50188929	G	A	-0.0359839	0.00431741	7.80E-17
rs139438549	16	67692863	T	C	-0.255605	0.0302117	2.70E-17
rs142507451	16	67694044	C	T	0.141513	0.0184333	1.60E-14
rs528301822	16	69403012	A	T	-0.0239697	0.00220584	1.70E-27
rs62053340	16	69987764	C	T	0.0213048	0.00207944	1.20E-24
rs34003787	16	73071381	C	T	0.0240518	0.00358352	1.90E-11
rs11866592	16	74654396	G	A	-0.03464	0.00286611	1.30E-33
rs2303262	16	82203758	C	T	0.0469485	0.00239917	2.90E-85
rs62046862	16	88073029	C	A	-0.0241905	0.002088	4.90E-31
rs9923119	16	90153815	T	C	-0.0176955	0.00240329	1.80E-13
rs7218033	17	1694247	C	T	0.022657	0.00230923	1.00E-22
rs4724	17	7760397	G	A	0.0547446	0.00312441	9.80E-69
rs75664430	17	8064779	C	G	0.0235179	0.0023186	3.60E-24
rs111527438	17	29252703	T	C	-0.0125	0.00211016	3.10E-09
rs12941945	17	41448228	A	G	0.0261033	0.00269093	3.00E-22
rs34405642	17	65741012	G	GA	-0.0128064	0.00214953	2.60E-09
rs2069536	17	74001106	A	G	0.0147561	0.00231358	1.80E-10
rs144204502	17	76183233	C	T	0.100574	0.00913369	3.40E-28
rs3891167	18	658423	A	G	0.0425685	0.00239551	1.20E-70
rs78694226	18	710980	G	A	0.0662878	0.0114386	6.80E-09
rs8088824	18	42151261	C	T	-0.0257674	0.00235716	8.10E-28
rs2276182	18	51798047	C	G	-0.0233529	0.00204247	2.80E-30
rs6565924	18	74691225	A	G	-0.0131327	0.00208399	2.90E-10
rs1879100	18	77985740	C	T	0.0191293	0.00294351	8.10E-11
rs80337039	19	4105089	G	T	-0.0814406	0.0136304	2.30E-09

© 2023 Aung N et al. *JAMA Cardiology*.

rs35601737	19	13220703	C	G	0.0140576	0.00220765	1.90E-10
rs8105767	19	22215441	A	G	-0.0328384	0.00220117	2.50E-50
rs4530278	19	33752994	G	T	-0.0138793	0.0020567	1.50E-11
rs11084431	19	56708667	G	A	0.0122304	0.00207797	4.00E-09
rs8102497	19	57370055	G	A	0.0149654	0.0020233	1.40E-13
rs1291143	20	35525640	A	C	-0.0493145	0.0027991	1.80E-69
rs544699357	20	41997057	C	T	-0.1691	0.0249084	1.10E-11
rs577449057	20	62236709	A	G	0.154143	0.0180647	1.40E-17
rs187013287	20	62298374	A	T	0.283074	0.0220182	7.90E-38
rs35640778	20	62321128	G	A	0.209011	0.00702087	9.60E-195
20:62321690_GAGA_G	20	62321690	GAGA	G	-0.118154	0.0174645	1.30E-11
rs115610405	20	62325833	C	A	0.10951	0.00733009	1.80E-50
rs187577818	20	62358869	A	C	-0.298357	0.0214698	6.60E-44
rs111527478	20	62678100	G	A	-0.02174	0.0033751	1.20E-10
rs28502153	22	17469049	C	A	0.0215916	0.00206208	1.20E-25
rs5845706	22	45779013	C	CA	-0.014823	0.0021116	2.20E-12
rs131796	22	50971639	G	GA	0.0243757	0.00236559	6.70E-25

EA, effect allele; NEA, non-effect allele; SE, standard error

© 2023 Aung N et al. *JAMA Cardiology*.

eTable 5. Mendelian Randomisation Results for the Association Between LTL and Cardiovascular Measurements and Heart Failure

Trait	method	Primary analysis				After Steiger filtering			
		nsnp	beta	95% CI	p value	nsnp	beta	95% CI	p value
LV mass	IVW	130	0.130	0.072 to 0.188	1.09E-05	121	0.118	0.066 to 0.171	1.03E-05
LV mass	Weighted median	130	0.127	0.034 to 0.22	7.52E-03	121	0.126	0.038 to 0.213	4.75E-03
LV mass	MR RAPS	130	0.134	0.08 to 0.187	8.84E-07	121	0.121	0.067 to 0.174	1.11E-05
LV mass	MR-Egger (Intercept)	-	0.000	-0.003 to 0.003	7.80E-01	-	-0.002	-0.005 to 0.001	1.49E-01
Global ventricular volume	IVW	130	0.078	0.016 to 0.14	1.35E-02	125	0.067	0.009 to 0.124	2.24E-02
Global ventricular volume	Weighted median	130	0.079	-0.01 to 0.169	8.30E-02	125	0.075	-0.013 to 0.163	9.44E-02
Global ventricular volume	MR RAPS	130	0.067	0.014 to 0.12	1.31E-02	125	0.059	0.006 to 0.113	2.91E-02
Global ventricular volume	MR-Egger (Intercept)	-	-0.001	-0.004 to 0.002	5.66E-01	-	-0.002	-0.005 to 0.001	1.47E-01
Overall ventricular size from shape model	IVW	119	0.037	0 to 0.074	4.90E-02	108	0.031	-0.001 to 0.063	5.89E-02
Overall ventricular size from shape model	Weighted median	119	0.036	-0.014 to 0.086	1.60E-01	108	0.034	-0.019 to 0.088	2.05E-01
Overall ventricular size from shape model	MR RAPS	119	0.035	0.004 to 0.066	2.59E-02	108	0.031	-0.001 to 0.062	5.65E-02
Overall ventricular size from shape model	MR-Egger (Intercept)	-	0.000	-0.002 to 0.002	9.12E-01	-	-0.001	-0.002 to 0.001	4.65E-01
LVSV	IVW	130	0.078	0.017 to 0.14	1.30E-02	122	0.054	-0.004 to 0.112	6.63E-02
LVSV	Weighted median	130	0.076	-0.011 to 0.163	8.67E-02	122	0.058	-0.031 to 0.146	2.00E-01
LVSV	MR RAPS	130	0.082	0.028 to 0.136	2.88E-03	122	0.060	0.006 to 0.114	3.05E-02
LVSV	MR-Egger (Intercept)	-	-0.001	-0.004 to 0.002	4.80E-01	-	-0.003	-0.006 to 0	6.45E-02
RVSV	IVW	130	0.058	-0.004 to 0.119	6.65E-02	124	0.044	-0.013 to 0.101	1.28E-01
RVSV	Weighted median	130	0.110	0.019 to 0.201	1.79E-02	124	0.109	0.023 to 0.194	1.30E-02
RVSV	MR RAPS	130	0.041	-0.013 to 0.094	1.41E-01	124	0.030	-0.025 to 0.084	2.84E-01
RVSV	MR-Egger (Intercept)	-	-0.002	-0.005 to 0.001	2.84E-01	-	-0.003	-0.005 to 0	9.02E-02
LA maximum volume	IVW	130	0.033	-0.027 to 0.093	2.75E-01	122	0.020	-0.037 to 0.077	4.92E-01
LA maximum volume	Weighted median	130	0.049	-0.043 to 0.141	2.96E-01	122	0.048	-0.046 to 0.143	3.16E-01

© 2023 Aung N et al. *JAMA Cardiology*.

LA maximum volume	MR RAPS	130	0.033	-0.025 to 0.091	2.60E-01	122	0.024	-0.034 to 0.083	4.18E-01
LA maximum volume	MR-Egger (Intercept)	-	0.002	-0.001 to 0.005	2.26E-01	-	0.000	-0.003 to 0.003	9.76E-01
LA emptying volume	IVW	130	0.025	-0.037 to 0.086	4.31E-01	124	0.021	-0.035 to 0.078	4.62E-01
LA emptying volume	Weighted median	130	0.080	-0.013 to 0.172	9.24E-02	124	0.080	-0.015 to 0.174	9.92E-02
LA emptying volume	MR RAPS	130	0.029	-0.029 to 0.087	3.35E-01	124	0.027	-0.031 to 0.086	3.58E-01
LA emptying volume	MR-Egger (Intercept)	-	0.001	-0.002 to 0.004	5.64E-01	-	0.001	-0.002 to 0.004	6.50E-01
Heart Failure	IVW	93	-0.044	-0.121 to 0.032	2.58E-01	93	-0.044	-0.121 to 0.032	2.58E-01
Heart Failure	Weighted median	93	-0.056	-0.17 to 0.058	3.34E-01	93	-0.056	-0.169 to 0.057	3.30E-01
Heart Failure	MR RAPS	93	-0.052	-0.123 to 0.019	1.52E-01	93	-0.052	-0.123 to 0.019	1.52E-01
Heart Failure	MR-Egger (Intercept)	-	0.000	-0.004 to 0.004	9.16E-01	-	0.000	-0.004 to 0.004	9.16E-01

MR, Mendelian Randomisation; IVW, inverse variance weighted; RAPS, Robust Adjusted Profile Score

© 2023 Aung N et al. *JAMA Cardiology*.
