# Supplementary material for: Association of Longer Leukocyte Telomere Length With Cardiac Size, Function, and Heart Failure
Source: JAMA Cardiol. 2023 Jul 26;8(9):808–15. doi: 10.1001/jamacardio.2023.2167 (PMC10372756; doi:10.1001/jamacardio.2023.2167)
Supplement: Supplement 2. — Data Sharing Statement [file jamacardiol-e232167-s002.pdf]

Data Sharing Statement

Aung. Association of Longer Leukocyte Telomere Length With Cardiac Size, Function, and Heart Failure. *JAMA Cardiol*. Published July 26, 2023. doi:10.1001/jamacardio.2023.2167

Data
Data available: Yes
Data types: Deidentified participant data
How to access data: ukbiobank.ac.uk/enable-your-research/apply-for-access
When available: With publication

Supporting Documents
Document types: None

Additional Information
Who can access the data: anyone requesting the data
Types of analyses: The Additional supporting information (statistical/analytic code) are available upon request.
Mechanisms of data availability: Email to the corresponding author
